# A Comprehensive Quality Evaluation Method Based on C_30_-HPLC and an Analytic Hierarchy Process for the Chinese Herbal Formula, Erzhiwan

**DOI:** 10.3390/molecules23082045

**Published:** 2018-08-15

**Authors:** Ying Nie, Weifeng Yao

**Affiliations:** 1 School of Pharmacy, China Pharmaceutical University, Nanjing 210009, China; nieyingcpu@163.com; 2 School of Pharmacy, Nanjing University of Chinese Medicine, Nanjing 210023, China

**Keywords:** analytic hierarchy process, C_30_-HPLC, Erzhiwan, herbal formula, quality evaluation

## Abstract

The quantitative analysis of multiple indexes remains an important quality evaluation method of traditional Chinese medicine (TCM) herbal formulas. The Chinese Pharmacopoeia 2015 only stipulates the content of a single component, specnuezhenide, in Erzhiwan composed of the Fructus Ligustri Lucidi (FLL) powder and aqueous extracts of Herba Ecliptae (HE). To generalize the intrinsic quality of Erzhiwan, a novel C_30_-HPLC method with good precision, accuracy, and reproducibility was developed for the simultaneous determination of six compounds, including two isomers, and then an analytic hierarchy process was further applied to integrate and discriminate the quality of four samples prepared via different methods. The results of the analysis were in agreement with the antioxidant tests in vitro. This comprehensive strategy could provide a reference and suggestions for the improvement of the quality evaluation method of TCM herbal formulas.

## 1. Introduction

As a combination of a range of herbal medicines based on specific clinical and practical experience, traditional Chinese medicine (TCM) herbal formulas sufficiently embody the therapeutic thought of syndrome differentiation over thousands of years. Unlike synthetic drugs, it is well known that herbal formulas generally exert their therapeutic effects through the synergic effects of multiple active ingredients and their multiple targets [1]. One of the key content of TCM quality evaluation is how to establish a multi-index quality control method that can effectively reflect the overall effect of TCM formulas [2]. However, the quantitative control of most TCM formulas in the Chinese Pharmacopoeia 2015 (ChP 2015) is only carried out using a single or a few indexes in the current quality evaluation. Erzhiwan (EZW), firstly recorded for nourishing liver and kidney in “Fu Shou Jing Fang” written in the Ming Dynasty, contains two herbs, Fructus Ligustri Lucidi (FLL) and Herba Ecliptae (HE) [3]. According to ChP 2015, it is composed of FLL (steamed) powder and aqueous extracts of HE, possessing the functions of tonifying the liver and kidney yin, strengthening tendon and bone, and arresting hemorrhage [4]. The main compounds from EZW, including phenylethanoid glycosides, iridoid glycosides, coumarins, triterpenes, and others, have antioxidant abilities [5,6,7,8,9], and EZW also plays many critical roles in protecting the liver and kidney [10,11]. However, the quality control of EZW still depends on the quantitation of the single-index specnuezhenide in the ChP 2015. In recent years, the simultaneous quantification of more components, such as salidroside, specnuezhenide, ligustroflavone, wedelolactone, oleanolic acid, and ursolic acid, was used to enhance the quality control of EZW [12,13,14,15,16]. The contents of these compounds are different in various samples; therefore, it is difficult to declare whether or not the sample is superior or inferior. Furthermore, chromatographic baseline separation could not be achieved using common reversed-phase columns for some isomeric compounds, such as oleanolic acid and ursolic acid, affecting the accuracy and reproducibility.

As a weighing methodological approach, the analytic hierarchy process (AHP) was developed for making effective decisions and achieving consensus from divergent judgments [17]. AHP could be better for solving the problems of the comprehensive evaluation of multi-component systems. It was widely used in method optimization, disease risk assessment, environment protection, and so on [18,19]. Recently, the AHP technique was also applied to the preliminary quality research of Chinese medicine [20]. As a novel reversed-phase column with a multilayer polymer stationary phase, the C_30_ HPLC column was firstly applied to separate isomeric carotenoids [21]. It was also widely used in improving the method for separating retinol [22], all-trans lycopene, and *β*-carotene [23]. Therefore, separation with a C_30_ column was more effective than that with a C_18_ column for non-polar geometric isomers [24].

In this study, six important compounds from the EZW formula, including two isomers, oleanolic acid and ursolic acid, were effectively separated and determined using C_30_-HPLC-UV. The antioxidant ability of each compound, tested using the 2,2-diphenyl-1-picrylhydrazyl (DPPH) method, were used for their weighting in the AHP. Combining the contents of the above indexes, the total scores were calculated for a comprehensive quality evaluation of a series of samples, including the traditional formula, EZW product (EZWP), and different compositions of FLL and HE, EZW-1, EZW-2, and EZW-3. The results of the AHP for all samples were further validated by the determination of antioxidant ability with the DPPH method. The main purpose of this study was to develop a comprehensive quality assessment method for TCM herbal formulas based on the content and efficacy of certain compounds.

## 2. Results and Discussion

### 2.1. Selection of the UV Wavelength for Determination

HPLC-UV was previously employed for the separation and determination of some of the six compounds in EZW. Specnuezhenide, wedelolactone, and oleanolic acid were detected at a wavelength of 215 nm using HPLC [13]. Since the UV absorbance wavelength of oleanolic acid and ursolic acid were at the terminal position of the spectrum, and their spectrum profiles were similar, the detective wavelength could be set at 215 nm (Figure S1E,F in the Supplementary Materials). Salidroside and wedelolactone in EZW were determined using an accurate HPLC method with a UV detective wavelength at 220 nm [12]. For better selecting the UV wavelength in HPLC, the UV spectra of salidroside, specnuezhenide, ligustroflavone, wedelolactone, oleanolic acid, and ursolic acid were also extracted from a photo diode array detector (Figure S1 in the Supplementary Materials). Therefore, the detective wavelength of the UV detector was set at 215 nm for the determination of all six compounds.

### 2.2. Selection of the Chromatography Column for Separation

**Figure 1:**
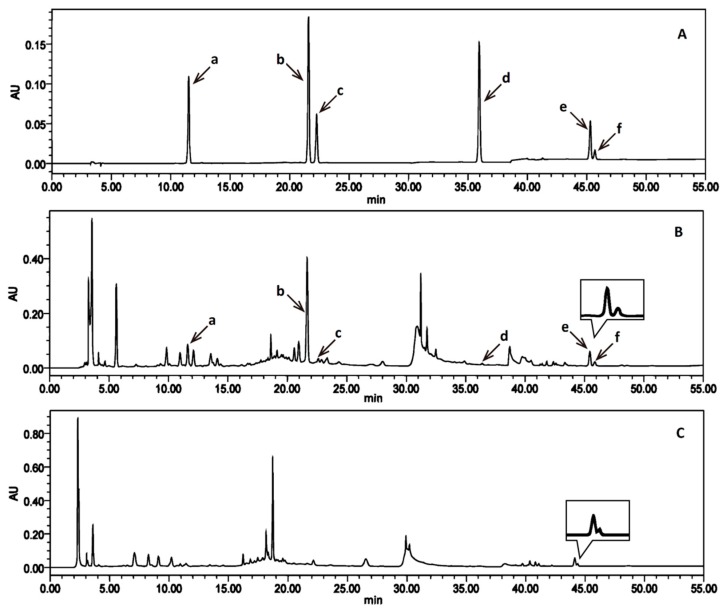
Chromatograms of reference substances using the C_30_-column (**A**); Erzhiwan (EZW)-3 sample using the C_30_-column (**B**); EZW-3 sample using the C_18_-column (**C**). Letters a–f represent salidroside (a), specnuezhenide (b), ligustroflavone (c), wedelolactone (d), oleanolic acid (e), and ursolic acid (f), respectively.

The resolution of oleanolic acid and ursolic acid using a C_18_ column was less than 1.5 (Figure 1C); thus, it could not be enrolled into the quantitation. As shown in Figure 1B, the resolution could reach 2.80 when the C_18_ column was replaced by a C_30_ column. It was revealed that the C_30_ phase exhibited superior resolution compared with the C_18_ phase in separating isomers. Compared with the C_18_ column, the C_30_ column with the multilayer polymer stationary phase formed a highly dense C_30_ alkyl chain on the surface of the silica gel to be more hydrophobic. Therefore, the C_30_ column had a higher retention and resolution of non-polar solutes.

### 2.3. Linearity, Range, Limit of Detection (LOD), and Limit of Quantitation (LOQ)

The standard solutions at six different concentrations were analyzed in triplicate. Table 1 shows the linear calibration regression equations, correlation coefficients, and the LOD and LOQ of salidroside, specnuezhenide, ligustroflavone, wedelolactone, oleanolic acid, and ursolic acid. S/N is defined as the power ratio of a signal (meaningful information) to the background noise (unwanted signal). The LOD is considered to be the concentration at which the S/N is equal to 3/1. The LOQ was the injection concentration corresponding to an S/N ratio of 10. The LODs and LOQs for all standard substances separated on the C_30_ column were confirmed, and showed that the method has sufficient sensitivity and precision.

### 2.4. Precision, Repeatability, and Stability

The precision was tested through continuous six-time determinations of the standard solution, and the values of relative standard deviation (RSD) were less than 0.96%. The repeatability was evaluated through six copies of determinations of the sample solution, and the RSD was less than 2.75%. Stability was assessed by analyzing three replicates of the same sample at different time intervals (0, 2, 4, 8, 12, 18, and 24 h), and the contents of the six compounds were found to be stable within 24 h (RSD < 2.88%, *n* = 3). All of these data in Table 1 indicate that the proposed method has high precision, reliable repeatability, and good stability.

### 2.5. Recovery

In order to determine the recovery, a known content of investigated compounds was added to the sample solution. Accurate amounts of each standard solution at equal concentrations were added to 0.5 g of EZW-3. The samples were prepared in sextuplicate using the same method. The mixture was extracted and analyzed. Good recoveries were obtained, and the results are also shown in Table 1. The mean recoveries of the investigated compounds ranged from 94.24 to 99.46%. The acceptable recovery indicates that the experimental protocol is feasible.

### 2.6. Assay of EZW Samples

**Figure 2:**
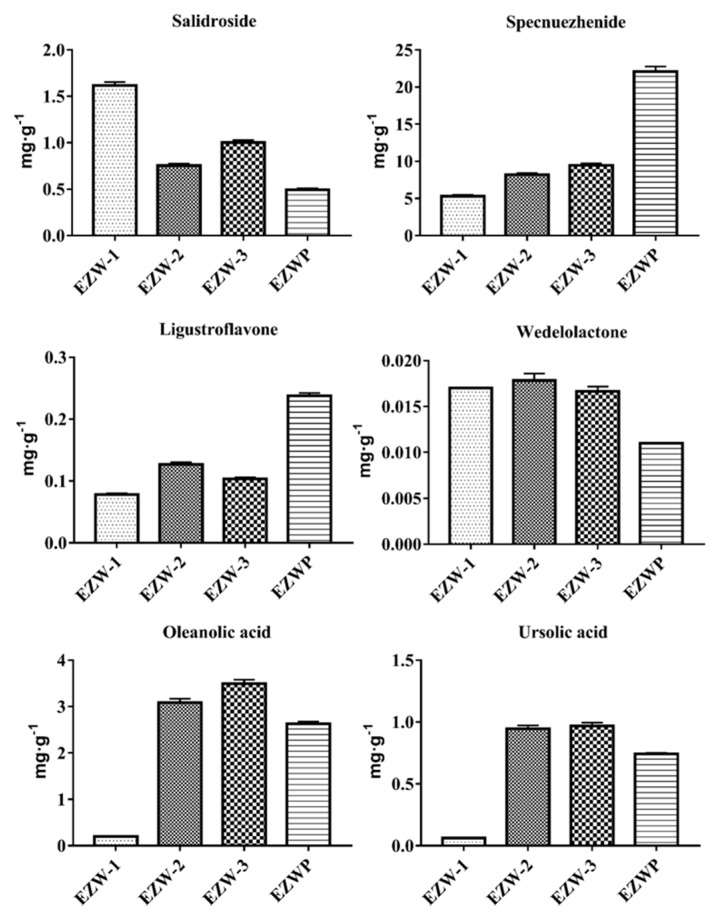
The contents of the six components from different EZW samples.

The HPLC method was subsequently applied to determine the contents of the six compounds in the samples. The quantitative determination results are shown in Figure 2. It demonstrates that there were distinct differences in the contents of the analytes from different EZW samples. All EZW samples met the requirements of the content of specnuezhenide prescribed by the ChP 2015. Compared with EZWP from the drug stores, the content of specnuezhenide was lower in EZW-1, EZW-2, and EZW-3. However, the contents of other compounds (salidroside, wedelolactone, oleanolic acid, and ursolic acid) in these samples were higher than in EZWP. These compounds cannot be ignored in the quality evaluation of EZW due to their good antioxidant activity [9,25,26,27]. The above results indicated that the quality control of a single index could not reflect the overall quality of the TCM herbal formula. Hence, the simultaneous determination of multiple indexes should be used in quality control. It is necessary to establish a novel comprehensive evaluation method for improving the quality control of EZW based on multicomponent quantification using C_30_-HPLC-UV.

### 2.7. AHP Analysis

As is known, the contents and activities of bioactive components are predominant for the drug’s effect [28]. Six components associated with the antioxidant function of EZW were selected for the AHP analysis, and the analytical results are shown in Table 2. The AHP analysis was carried out using the following steps. Firstly, the antioxidant activities of the components were measured using DPPH, and the half maximal inhibitory concentrations (IC_50_) were used as indicators to compare their relative pairwise importance (Table 3). Secondly, based on the IC_50_ values and the literature [29], the antioxidant activity of each standard was arranged in the following order: wedelolactone > oleanolic acid = ursolic acid > salidroside > specnuezhenide > ligustroflavone. Thirdly, the importance ratio of each component, making up the pairwise comparison matrix, was assigned. Fourthly, the consistency ratio (CR) value of the above matrix was calculated to detect its consistency. The result was 0.011 (<0.10), indicating that the consistency of the comparison matrix was acceptable. Finally, the comprehensive evaluation scores of the EZW samples were calculated. The score of EZW-3 was the highest with 0.837, while EZW-2 and EZW-1 acquired the second and third highest scores, with 0.781 and 0.463, respectively. EZWP tied in last place with 0.432. The scores of EZW-1, EZW-2, EZW-3, and EZWP were different, indicating that the AHP could distinguish and evaluate their quality.

### 2.8. Validation of the AHP Results In Vitro

The AHP result was a predictive theoretical result, requiring a verification of credibility. The antioxidant activity of EZW samples in vitro was used to test the credibility of the AHP results. The IC_50_ values of the EZW samples (Table 3), calculated using different clearance rates (Table S3), demonstrated that different antioxidant effects may be related to the different contents of the constituents in samples. Since a lower IC_50_ value represents higher antioxidant activity, the antioxidant activity of EZW samples was as follows: EZW-3 > EZW-2 > EZW-1 > EZWP. The results were consistent with the results of AHP analysis, indicating that the established AHP method of evaluating the EZW comprehensive quality was convincing.

## 3. Material and Methods

### 3.1. Materials and Chemicals

The samples of FLL and HE were purchased from HETIAN Chinese medicine Co. Ltd. (Tongling, China) and identified by Professor Qinan Wu (a TCM identification expert at Nanjing University of Chinese Medicine). Erzhiwan product (EZWP, Lot: 160901, Fuzhou, China) was acquired from Jiangxi Renfeng Pharmaceutical Co., Ltd. Furthermore, 2,2-diphenyl-1-picrylhydrazyl (DPPH) was bought from TCI Shanghai Development Co., Ltd. (Shanghai, China). The reference standards of salidroside, specnuezhenide, ligustroflavone, wedelolactone, and oleanolic acid were purchased from PUSH biotechnology Co. Ltd. (Chengdu, China) The reference standard of ursolic acid was bought from the National Institute for Food and Drug Control of China (Beijing, China). Acetonitrile and methanol were obtained from Merck (Darmstadt, Germany). Phosphoric acid was provided by Aladdin (Shanghai, China). Deionized water was obtained from a Milli-Q system (Millipore, Bedford, MA, USA).

### 3.2. Apparatus

Quantitative HPLC was performed on a Waters 2695 with a 2996 photo diode array detector HPLC system (Waters, MA, USA), and a personal computer (PC) with the Empower work station for data processing. Agilent provided an Extend-C_18_ column (250 mm × 4.6 mm i.d., 5-μm particle size, Santa, Clara, CA, USA), and Welch provided an Ultimate^®^XB C_30_ column (250 mm × 4.6 mm i.d., 5-μm particle size, Shanghai, China). Ultrasound-assisted extraction (UAE) was performed using an ultrasonic cleaning bath (KQ-250V, Kun-Shan Ultrasonic Instruments Co., Ltd., Kunshan, China). The absorbance was measured at 517 nm for the microplate reader (Infinite^®^Pro200 TECAN, Männedorf, Switzerland).

### 3.3. Preparation of the Stock Solution

The standard stock solutions of salidroside (0.234 mg·mL^−1^), specnuezhenide (1.502 mg·mL^−1^), ligustroflavone (0.113 mg·mL^−1^), wedelolactone (0.079 mg·mL^−1^), oleanolic acid (0.576 mg·mL^−1^), and ursolic acid (0.168 mg·mL^−1^) were prepared in 80% methanol and then stored away from light at 4 °C. Working solutions were prepared using the appropriate dilution of the obtained stock solution.

### 3.4. Preparation of EZW Samples

Wine FLL was the steamed product of FLL mixed with wine [30]. For the preparation of EZW, it was dried and ground into powder in accordance with the ChP 2015. The extracts of wine FLL were prepared separately by extracting the powders with water or 95% alcohol. For the aqueous extract (or alcohol extract), 100 g of wine-FLL powder was soaked and boiled in 1000 mL of water (or 95% alcohol) under reflux twice, for two hours each time. For extract A of wine FLL, 100 g of powder was soaked and boiled in 1:10 (*w*:*v*) 95% alcohol twice, and for extract B, the filter residue was boiled in 1:10 (*w*:*v*) water twice, for two hours each time. Following the above steps, the solutions of each extract were filtered, condensed, and dried by lyophilization. The aqueous extract of HE was prepared according to the ChP 2015. EZW-1 was made up of aqueous extracts of HE and wine FLL according to their crude-drug ratio. EZW-2 contained an aqueous extract of HE and an alcohol extract of wine FLL. EZW-3 was composed of extracts A and B of wine FLL, and an aqueous extract of HE in the same crude-drug ratio. All samples were stored at −20 °C. The herbal extraction was done on three independent batches of herbs, and the voucher specimen was deposited in the Level-3 Laboratory for the Chinese Medicine Chemistry of State Administration of TCM at the Nanjing University of Chinese Medicine.

### 3.5. Sample Preparation for HPLC

The above samples were appropriately ground to powder, and the powder (1.0 g) was mixed with 25 mL of 80% methanol in a 50-mL conical flask with a cover. The mixture was extracted ultrasonically (40 kHz, 600 W, 25 °C) for 30 min. After cooling, the solution was then made up to the required volume. Then, the mixture was centrifuged for 10 min at 15,000 rpm to deposit the suspension particle, and the supernatant was injected into the HPLC system.

### 3.6. Chromatographic Conditions

For the quantitation of salidroside, specnuezhenide, ligustroflavone, wedelolactone, oleanolic acid, and ursolic acid, HPLC was conducted using a Welch Ultimate^®^XB C_30_ column (250 mm× 4.6 mm i.d., 5-μm particle size), and the mobile phase consisted of acetonitrile (solution A) and 0.1% phosphoric acid in water (solution B). The flow rate, column temperature, and injection volume were set to 1 mL·min^−1^, 30 °C, and 10 μL, respectively. The gradient program was set as follows: 0–5 min with 5–9% A, 5–12 min with 9–14% A, 12–15 min with 14–23% A, 15–26 min with 23% A, 26–27 min with 23–35% A, 27–35 min with 35–40% A, 35–36 min with 40–91% A, 36–48 min with 91% A, 48–50 min with 91–95% A, 50–52 min with 95% A, 52–55 min with 95–5% A. The wavelength of the UV detector was fixed at 215 nm for determination.

### 3.7. Antioxidant Activity In Vitro

The DPPH method is widely used for testing the antioxidant activity of compounds in vitro. The antioxidant activity of each compound and of various EZW samples was determined using the DPPH radical scavenging method according to the method outlined by Majeed et al. [31]. The DPPH working solution (0.041 mg·mL^−1^) was prepared by dissolving and diluting the powder with methanol, and the stock solutions of salidroside, specnuezhenide, ligustroflavone, wedelolactone, oleanolic acid, and ursolic acid were prepared in 80% methanol to 1.04, 1.08, 1.02, 0.206, 0.232, 0.222 mg·mL^−1^, and then stored away from light at 4 °C. Sample solutions were prepared using the appropriate dilution of the obtained stock solution. The solution of 90% methanol was the blank group, and the methanol and sample solutions were mixed with equal volumes of DPPH working solution as the control groups and test groups. After an incubation period of 30 min in the dark, the absorbance was read at 517 nm. All tests were repeated three times, and the results were substituted into the Equation (1) to calculate the scavenging rate (SR). The concentration of the sample scavenging 50% of the DPPH free radicals was named the IC_50_ value, and a smaller IC_50_ value represented a higher antioxidant activity. The IC_50_ value of each sample was calculated using the SPSS 20.0 software (SPSS, Chicago, IL, USA).

(1) $$\mathrm{SR}/\%=(1-\frac{A_{3}-A_{0}}{A_{1}})\times 10,$$

where A_0_, A_1_, and A_2_ are the absorbance values of the blank, test, and control, respectively.

### 3.8. Analytic Hierarchy Process

Firstly, six compounds (salidroside, specnuezhenide, ligustroflavone, wedelolactone, oleanolic acid, and ursolic acid) with antioxidant activity were chosen as the indexes for evaluating the quality of the EZW samples. Then, according to their radical scavenging activity, the relative pairwise importance of two indexes was obtained from a nine-point numerical scale (Table S1 in the Supplementary Materials) to indicate the importance ratio of one attribute to another [32]. The data consisting of a pairwise comparison matrix were used to find the comparative weights among the attributes of the decision elements. There was some subjectivity in that the generated pairwise comparison matrix was from the importance ratio of one attribute to another. Because the comparison matrix could not become a positive reciprocal matrix, it could be allowed to have some degree of inconsistency. In order to ensure the consistency of the subjective perception and the accuracy of the comparative weights, the consistency index (CI) and the consistency ratio (CR) were calculated using the equations below. If the CR was lower than or equal to 0.10, it was considered acceptable. If it was higher than 0.10, the importance ratio was judged again [17].

(2) $$w_{i}^{\prime }=\sqrt[{m}]{a_{i1}a_{i2}\cdots a_{\mathrm{im}}},$$

(3) $$w_{i}=\frac{w_{i}^{\prime }}{\sum_{i=1}^{m}w_{i}^{\prime }},$$

(4) $$\lambda_{\max}=\frac{\sum_{i=1}^{m}\left(\sum_{j=1}^{m}\frac{\left(a_{\mathrm{ij}}w_{j}\right)}{w_{i}}\right)}{m},$$

(5) $$\mathrm{CI}=\frac{\lambda_{\max}-m}{m-1},$$

(6) $$\mathrm{CR}=\frac{\mathrm{CI}}{\mathrm{RI}},$$

where a, i, j, and m are the elements, columns, rows, and order of the judgment matrix, respectively. $w_{i}^{\prime }$, $w_{i}$ are the separated initial weights and normalized weights. $\lambda_{\max}$ is the largest eigenvalue for the judgment matrix. The random index (RI) is the mean randomness consistency of the comparison matrix. For a 6 × 6 matrix, its value was 1.24 queried from Table S2.

The ultimate purpose of this comprehensive evaluation was to synthesize all of the index values into an integrality evaluation score, after which we could rank different EZW samples. The raw data of each criterion were standardized in order to rule out the effects of different dimensions and the disparities in orders of magnitude. Considering the nature of the methods of standardization and the possible ranges in data after standardization, we performed calculations using the following equation (the min–max normalization) to standardize the raw data of the criteria:

(7) $$x_{\mathrm{ij}}=\frac{a_{\mathrm{ij}}-m_{j}}{M_{j}-m_{j}},$$

where M_j_ and m_j_ are the results of the max {a_ij_} and min {a_ij_}.

Finally, to meet the study aims, a linear weighting method was chosen as the method of determining the criterion weights, as it was appropriate for comprehensively evaluating synthetic criterion data.

(8) $$y=\sum_{i=1}^{m}w_{i}x_{i},$$

where w_i_ is the corresponding weight of x_i_ (0 ≤ x_i_ ≤ 1 (i = 1, 2, …, m, $\sum_{i=1}^{m}w_{i}$ = 1), x_i_ is the value of each evaluation criterion, and y is the comprehensive evaluation score, the value of which is based on the quality. A higher score represented a better sample quality in the evaluation system.

## 4. Conclusions

In this work, a comprehensive strategy using C_30_ HPLC coupled with AHP analysis was proposed for the quality evaluation of the TCM formula, EZW. The evaluation data of the AHP analysis were completely consistent with the antioxidant activity test using DPPH in vitro. The above results revealed that it was feasible to use this comprehensive strategy for the quality evaluation of EZW. The developed quantitative method is promising for improving the quality evaluation standard of EZW by determining multiple indexes. Therefore, this strategy also has great guiding significance for the quality evaluation of TCM formulas according to the pharmacological activities and contents of the measured indexes.

## Figures and Tables

**Table 1:** Calibration curves, limits of detection (LODs), limits of quantitation (LOQs), precision, repeatability, stability, and recoveries of the six analytes.

Component	Calibration Curves	*r*	Linear	LOD	LOQ	RPrecision	Repeatability	Stability	Recoveries *	
			(μg·mL^−1^)	(μg·mL^−1^)	(μg·mL^−1^)	RSD ^#^	RRSD	RRSD	(%, *n* = 6)	
						(%, *n* = 6)	R(%, *n* = 6)	(%)	Mean	RSD
Salidroside	*y* = 16,251*x* − 475.66	0.9996	0.46–234.00	0.034	0.110	0.92	1.81	2.88	95.17	1.71
Specnuezhenide	*y* = 8414.3*x* − 524.10	0.9997	1.47–1502.00	0.064	0.210	0.12	1.06	1.73	98.15	2.30
Ligustroflavone	*y* = 21,308*x* − 781.94	0.9995	0.22–113.00	0.028	0.094	0.31	2.38	2.46	99.05	0.47
Wedelolactone	*y* = 72,542*x* − 3010.1	0.9994	0.15–79.00	0.009	0.028	0.18	2.17	2.18	99.43	0.72
Oleanolic acid	*y* = 3259.*9x* − 1509.8	0.9995	2.25–576.00	0.200	0.650	0.37	2.41	0.70	94.24	1.91
Ursolic acid	*y* = 2800.5*x* − 1339.7	0.9998	1.31–168.00	0.250	0.840	0.96	2.75	1.34	94.66	1.87

* Recoveries (%) = (found amount − original amount)/spiked amount × 100. ^#^ RSD: relative standard deviation.

**Table 2:** Decision matrix of the pairwise comparison of indexes and results of the multi-index weight.

	Salidroside	Specnuezhenide	Ligustroflavone	Wedelolactone	Oleanolic Acid	Ursolic Acid	λ_max_	CI	CR	w_i_’	w_i_
Salidroside	1	2	3	1/3	1/2	1/2	6.072	0.014	0.011	0.891	0.122
Specnuezhenide	1/2	1	2	1/4	1/3	1/3				0.550	0.075
Ligustroflavone	1/3	1/2	1	1/5	1/4	1/4				0.357	0.049
Wedelolactone	3	4	5	1	2	2				2.493	0.341
Oleanolic acid	2	3	4	1/2	1	1				1.513	0.207
Ursolic acid	2	3	4	1/2	1	1				1.513	0.207

Notes: λ_max_ is the largest eigenvalue for the judgment matrix. CI is the consistency index. CR is the consistency ratio. w_i_’ is the initial weights. w_i_ is the normalized weights.

**Table 3:** Half maximal inhibitory concentrations (IC_50_) of various standard stock and Erzhiwan (EZW) samples (*n* = 3).

	**IC_50_ (mg·mL^−1^)**
Salidroside	1.640
Specnuezhenide	2.125
Ligustroflavone	3.660
Wedelolactone	0.045
Oleanolic acid	0.321
Ursolic acid	0.324
	**IC_50_ (mg·mL^−1^)**
EZW-1	0.357
EZW-2	0.321
EZW-3	0.224
EZWP	0.518

## Supplementary Materials

Supplementary materials Figure S1, Tables S1–S3 are available online.
