# Supplementary material for: A Comprehensive Quality Evaluation Method Based on C30-HPLC and an Analytic Hierarchy Process for the Chinese Herbal Formula, Erzhiwan
Source: Molecules. 2018 Aug 15;23(8):2045. doi: 10.3390/molecules23082045 (PMC6222504; doi:10.3390/molecules23082045)
Supplement: Supplementary file 1 [file molecules-23-02045-s001.pdf]

A Comprehensive Quality Evaluation Method Based on C30-HPLC and an Analytic Hierarchy Process for the Chinese Herbal Formula, Erzhiwan

Ying Nie[1], Weifeng Yao[2]*.

1. School of Pharmacy, China Pharmaceutical University, Nanjing 210009, China; nieyingcpu@163.com (Y.N.)
2. School of Pharmacy, Nanjing University of Chinese Medicine, Nanjing 210023, China;

   yaowf@njucm.edu.cn (W.F.Y)

**Corresponding Author*: yaowf@njucm.edu.cn (W.F.Y)
Tel.: +86-25-85811053 (W.F.Y)

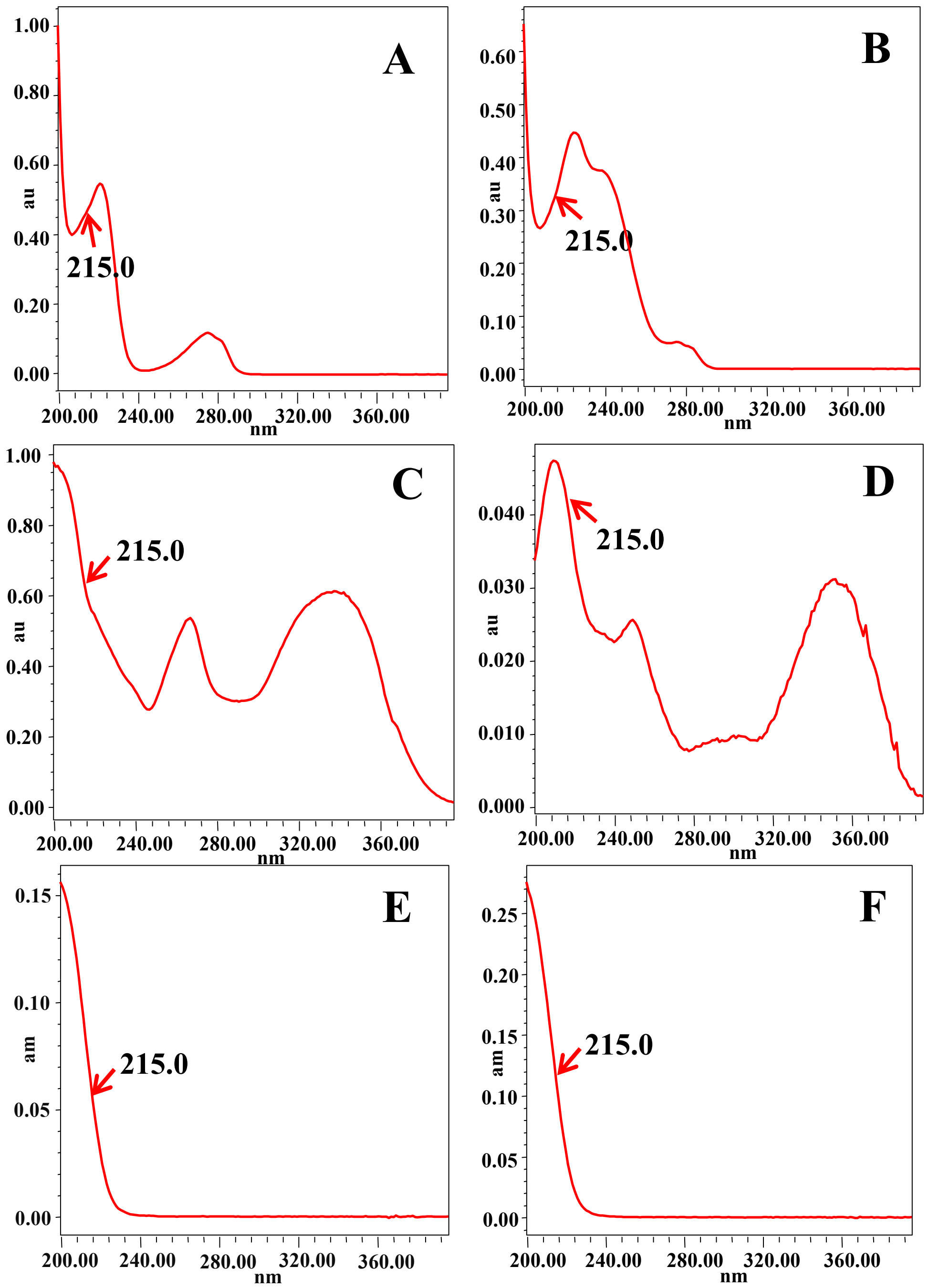

Figure.S1. The UV spectrums of salidroside (A), specnuezhenide (B), ligustroflavone (C), wedelolactone (D), oleanic acid (E) and ursolic acid (F).

Table S1. Pair comparison evaluation scale.

Importance	Definition	Explanation
1	Equal importance	Two activities contribute equally to the subject
3	Weak importance	One activity is slightly more important than the other
5	Essential importance	One activity is exceedingly more important than the other
7	Demonstrated importance	One activity has been practically demonstrated as more important.
9	Absolute importance	Evidence favors one activity as more important than the other of the highest possible order of affirmation
2, 4, 6, 8	Intermediate values	When compromise is needed

Table S2. Random index for consistency analysis of expert raters.

N	3	4	5	6	7	8	9
RI	0.58	0.90	1.12	1.24	1.32	1.41	1.45

Where N is the order of comparison matrix.

Table S3. The DPPH clearance rate of EZW samples at different concentrations (n=3, %).

	0.01 $mg\cdot mL^{-1}$	0.04 $mg\cdot mL^{-1}$	0.16 $mg\cdot mL^{-1}$	0.63 $mg\cdot mL^{-1}$	2.50 $mg\cdot mL^{-1}$
EZW-1	25.77	31.02	45.56	82.67	93.14
EZW-2	29.66	30.45	47.26	81.91	93.83
EZW-3	33.58	34.19	51.29	83.43	93.72
EZWP	27.53	32.07	39.16	69.79	89.62
